# Effects of continuous cover management on bird communities in a beech dominated forest region of Slovenia

**DOI:** 10.1038/s41598-025-19071-x

**Published:** 2025-09-29

**Authors:** Małgorzata Bujoczek, Leszek Bujoczek, Thomas A. Nagel

**Affiliations:** 1 Department of Forest Biodiversity, University of Agriculture in Krakow, Faculty of Forestry, Al. 29 Listopada 46, Krakow, 31-425 Poland; 2 Department of Forest Resources Management, University of Agriculture in Krakow, Faculty of Forestry, Al. 29 Listopada 46, Krakow, 31-425 Poland; 3 Department of Forestry and Renewable Forest Resources, Biotechnical Faculty, University of Ljubljana, Ljubljana, Slovenia

**Keywords:** Old-growth forest, Managed stands, Cavity-nesters, Old trees, Habitat’s mosaic, Biodiversity, Ecosystem ecology, Ecosystem services, Forest ecology, Forestry

## Abstract

Forest management can have a negative impact on the populations of some forest species. One solution has been to use management that is better at mimicking natural processes. However, the effectiveness of such systems for maintaining some taxa needs further research. We evaluated forest structure in European beech (*Fagus sylvatica* L.) dominated stands managed with uneven-aged, continuous cover silviculture, compared to an old-growth stand, and characterized their bird communities. The managed stands represented different developmental stages of the forest, including a mature stand (before regeneration cutting), a gap-cut stand (a mature stand in the process of partial cutting), and a young stand. The old-growth stand had many features distinguishing it from the managed stands. In the managed stands, tree diameter reached a maximum of 67 cm in the gap-cut stand, and the highest mean stand volume was 459 m^3^ ha^− 1^ in the mature stand. In the old-growth forest stand, the volume was 807 m^3^ ha^− 1^ and there were 24 trees ha^− 1^ larger than 67 cm in DBH. These large stems alone had a volume of more than 300 m^3^ ha^− 1^. Additionally, the managed stands had a deadwood volume in the range of 19–25 m^3^ ha^− 1^, mainly consisting of stumps and small diameter fragments, compared to 318 m^3^ ha^− 1^ in the old-growth forest. The number of bird species was linked to the number of niches available, resulting from the presence of large-diameter trees, a heterogeneous diameter at breast height structure, and varying densities in the lower forest layers, which was reflected in the density of breeding pairs, from 104 per 10 ha in the old-growth forest to 22–87 pairs per 10 ha in the managed stands. In the breeding season the Shannon index for the old-growth forest was 4.29 versus 2.81–3.94, in the young and gap-cut stand, respectively. Half of the species found in the old-growth forest nested in cavities. Rare, more specialized species occurred more often in the old-growth forest. The presence of a dynamic mosaic of forest development stages turned out to be important for the diversity of bird species. However, the presence of large old trees and deadwood are likely key factors for maintaining bird diversity. Thus, management practices should be modified to better focus on the protection of microhabitats supporting the species associated with them.

**Supplementary Information:**

The online version contains supplementary material available at 10.1038/s41598-025-19071-x.

## Introduction

Understanding the relationship between species requirements and habitat characteristics at both local and landscape scales is an important step towards guiding forest management toward approaches that better maintain native biodiversity^1–3^. The presence of animals in a given area is determined by the availability of habitats of appropriate quality^4^. In forest ecosystems, habitat quality at stand scales is largely a function of forest species composition and structure, such as the spatial arrangement of trees and their size diversity, canopy openings that influence understory microclimate, and dead and dying trees (e.g. snags, coarse woody debris, and veteran trees)^5,6^. Disturbances in forest ecosystems, including agents that are both natural (e.g. windthrow and insect outbreaks) and anthropogenic (e.g. timber harvesting), exert strong control on forest structure, composition, and the biodiversity within them^7,8^. This also applies to avifauna, which may respond positively or negatively to changes in forest structure and composition^9^.

In Europe, one of the most important forest types includes beech (*Fagus sylvatica)* dominated forests, which in the absence of widespread historic land use, would be the dominant habitat cover in Central Europe, and with which a large number of animal species are associated^10^. Due to the diversity of environmental and climatic conditions, different community types of beech forest occur in the region^11^. They range from monospecific beech forests to multi-species communities featuring other coniferous and/or deciduous species, and include both lowland and mountain forests where beech is the most dominant deciduous species^12,13^. Due to the widespread occurrence of beech forests, nearly all of them constitute an important part of managed forests and have been subject to significant transformations in terms of structure. The main silvicultural system currently in use is the regular shelterwood system^14^, which results in naturally regenerated even-aged stands, and a mosaic of stand ages at larger landscape scales. This patchwork of different aged stands has been found to support a wide range of biodiversity at the landscape scale^15^.

When left unmanaged, beech forests have a tendency to develop into multi-layered, multi-aged stands due to a combination of gap-phase dynamics and moderate severity natural disturbances^12,14,16^. The more coarse-scaled even-aged patches created by shelterwood management differ substantially from the disturbance processes and resulting structures found in unmanaged forests, where natural processes typically lead to the development of old-growth development stages in the absence of large-severe disturbances^14,17^. For example, old-growth stands contain large amounts and diverse types of deadwood, legacies from natural disturbances, and large, old trees with a rich diversity of microhabitats^18–20^. Using systems of continuous cover forestry or natural disturbance based silviculture, it is possible to maintain more complex forest structures associated with old-growth beech forests^14^, but not all of its components. For example, in forests where wood production is a primary goal, both more conventional shelterwood systems and uneven-aged systems mimicking natural disturbance processes will not maintain the same amount and quality of deadwood found in old-growth stands^21,22^. Consequently, managed forests may not provide suitable habitat for all organisms associated with beech forests, particularly more demanding or specialized species that depend on old-growth habitat structures, such as large amounts of high quality deadwood or rare microhabitats^23^.

In Slovenia, forests cover about 60% of the land area and are managed in accordance with close-to-nature management principles, such as reliance on native tree species and natural regeneration, and the use of relatively fine-grained, continuous cover silvicultural systems without clear-cutting^24–26^. Additionally, forest regulations require that coarse woody debris (CWD) make up at least 3% of total wood volume. Due to the relatively well-preserved forests, as much as 45% of Slovenian forests is designated as Natura 2000, helping to ensure maintenance of key species and habitats for the region and the EU (e.g. large predators, Alpine Longhorn Beetle (*Rosalia alpina* L.), Jersey Tiger Moth (*Callimorpha quadripunctaria* Poda), White-backed Woodpecker (*Dendrocopos leucotos* Bechstein, JM), European Scopolia (*Scopolia carniolica* Jacq.), and many others). In principle, this method of forest management fulfills both economic and ecological functions, such that strictly protected forest reserves cover less than 1% of the total forest area^22^. There are 164 national forest reserves in Slovenia, but most of them are small (< 20 ha), and in some stage of recovery from past timber extraction. Sixteen of them, collectively representing 0.07% of the total forest area, are classified as old-growth^27^. The concurrent fulfillment of economic and ecological functions requires more rigorous validation, and this research forms part of this effort.

Using an old-growth stand as a reference, we ask how the traditional form of close-to-nature silviculture in beech forests of the study region influences bird assemblages. While it is known that some specialist bird species are linked to structures found in old-growth conditions (e.g. the white backed woodpecker, *Dendrocopos leucotos*) [e.g. ^28,29^], it is less clear how stand structures derived from continuous cover, uneven-aged silvicultural systems affect bird communities. To answer this question, the paper presents a detailed description of forest structural indicators (e.g. living trees, deadwood, and regeneration) and bird assemblages both in breeding and non-breeding seasons in an old-growth forest stand and in nearby managed stands. The managed stands clearly differed in age and size structure, capturing a range of developmental stages present within the managed forests of the study region. We aimed to (1) quantify the diversity of structural conditions in beech forests managed with continuous cover silviculture compared with an old-growth stand; (2) determine the species composition of bird communities and the density of breeding pairs; (3) define ecological indicators for bird assemblages in the non-breeding season; and (4) explore the relationship between forest structural indicators and bird diversity.

## Study area and field methods

### Study area

**Fig. 1:**
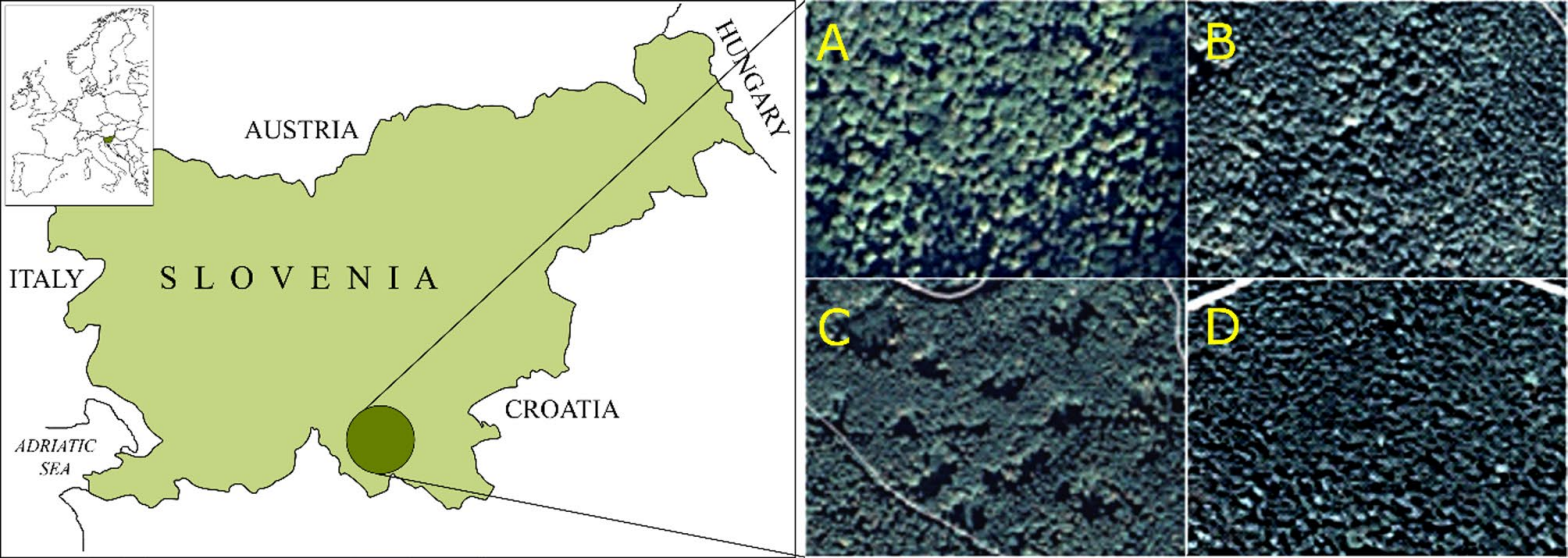
Study area and the four studied sites: (**A**) old-growth Pečka Reserve (45° 45’ 16.8” N 14° 59’ 37.8” E); (**B**) mature stand (before regeneration cutting, 45° 36’ 02.0” N 15° 00’ 44.9” E); (**C**) gap-cut stand (mature stand in the process of partial cutting, 45° 36’ 28.7” N 15° 00’ 22.1” E); (**D**) young stand (45° 46’ 17.9” N 15° 00’ 46.2” E) [Map screenshot, Google Maps, https://www.google.com/maps; software Inkscape 0.92.4, https://inkscape.org/].

Research was conducted in forests located in the Kočevsko-Kolpa Important Bird Area, which is an extensive area of ​​limestone karst on the Dinaric mountain plateau in southern Slovenia (Fig. 1). This IBA consists of coniferous, deciduous, and mixed forests. Together with the Snežnik plateau and Gorski Kotar in Croatia, it represents one of the largest and least populated areas of dense forests in Europe. Forests covers 95% of the area, including some remnants of old-growth forest, while pastures and grasslands account for 5%^30^.

The study was carried out in 2021–2022 on 4 selected sites with an area of ​​30 ha each (3 managed stands and 1 old-growth reserve), where the dominant species was beech. These stands were adjacent to other forest stands exhibiting a species composition comparable to that of the study sites. The sites are located at an elevation of approx. 500 (young stand) and 800–900 m a.s.l. (remaining stands) and feature a combination of maritime and continental climates. They are characterized by warm summers, cold and snowy winters, and abundant rainfall (> 1500 mm/year), especially in spring and autumn. The microtopography of the sites is very diverse, with numerous sinkholes typical of karst geology. Calcareous brown soils on the sites are derived from the limestone parent material.

The old-growth Pečka Reserve, now under strict protection, was selected as a reference stand, to which the stand characteristics and bird communities of three managed stands at different stages of forest development were compared. These stands form part of a larger landscape level mosaic formed from uneven-aged, continuous cover silviculture used in the region, which is mainly the irregular shelterwood system. This system includes a combination of low intensity shelter cuts (removal of individual trees systematically over mature stands to promote regeneration) and creation of gaps typically not larger than a stand height in diameter distributed over the stand to continue the recruitment of regeneration into upper canopy layers. At the same time, high quality trees in the upper canopy are released for diameter growth. The three stands included: 1). a mature stand (before regeneration cutting); 2). a gap-cut stand (a mature stand in the process of partial cutting); and 3). a young stand. Structural differences among the stands are evident in the DBH distributions presented in Appendix A, Figure A1, A. It is important to note that the irregular shelterwood system has been practiced for about the past 70 years at the study site, but many older stands (> 100 years) are still rather homogeneously structured as a result of more regular shelterwood approaches used over a century ago. However, these three managed sites capture the range of forest structures typical for irregular shelterwood in this region. On all sites, birds counts and stand measurements, including both live and dead trees, were performed.

### Stand measurements

A total of 100 sample plots were established (25 per stand) to quantify various structural attributes. In each of the four studied stands, the sample plots were placed on grid nodes measuring 100 × 100 m. The selection of sample plot dimensions, classification criteria for tree layer, saplings, and seedlings, and the approach to deadwood description were informed by methodologies established in earlier forest inventory research [e.g. ^9,31,32^]. The sample plots were 0.04 ha for trees and deadwood, 0.01 ha for saplings, and 4 × 1.13 m^2^ for seedlings. We identified the species and measured the diameter at breast height (DBH) of all live and dead trees (DBH ≥ 7 cm) on each sample plot (Table 1). The heights of trees located within the boundaries of a 0.01 ha circular plot were measured with a Vertex IV. Additional measurements were taken for stumps and downed deadwood, including diameters and decay stages (Table 1). The applied classification of decay stages closely follows the system proposed by Maser et al.,^33^. Saplings were classified into to one of three size classes (0.5 m ≤ height < 1.3 m; height ≥ 1.3 m and DBH < 4; and 4 ≤ DBH < 7 cm). Seedlings were defined as having a height of up to 0.5 m. On each sample plot (0.04 ha), canopy closure was visually assessed as a percentage of ground area shaded by overhead foliage.

**Table 1:** Deadwood classification and measurement.

Measured characteristic	Rules for measurement and evaluation of characteristics
Deadwood type	
Standing entire (complete) dead trees (including crown) with DBH ≥ 7 cm	DBH and height
Snags – standing snapped trees with height ≥ 1.3 m and DBH ≥ 7 cm)	Height and three diameters: DBH, above ground level, and at the top of the snag
Stumps with height < 1.3 m and diameter above the ground ≥ 10 cm	Height and two diameters: above ground level and at the top of the stump
Downed deadwood Fallen logs, trunks or branch fragments (pieces of trunk or large branches, etc.)	Diameters at the end of each deadwood piece were measured and the length of the piece was recorded (pieces with a diameter of less than 7 cm were excluded)
Deadwood decay stage (recorded on a 5-point scale for both lying and standing deadwood)	
Decay stage I	Round shape, bark present or missing only in places, wood of original colour, hard surface, thick and thin branches present; logs raised above the ground, resting on branches
Decay stage II	Round shape, bark often peeled off on the underside of lying logs and completely missing on standing deadwood, wood surface smooth, slightly bends under the pressure of a sharp tool, thicker branches preserved
Decay stage III	Round shape, non-adherent bark easily torn off by hand, wood with visible furrows up to about 1 cm, wood fibrous and soft, wood can be penetrated by a sharp object up to a depth of 3–5 cm, fragments can be easily removed by prying, only the base parts of branches present, logs partially rest on the surface of the soil
Decay stage IV	Oval shape, no bark or bark lies loosely on the wood, wood with clear furrows, hard only in the central part; larger hard fragments can be easily removed with a sharp tool, logs closely adhere to the ground, often almost completely overgrown by vegetation
Decay stage V	Forms a ridge raised above the surrounding terrain, closely adheres to the ground, reflecting any unevenness, fibrous or unstructured wood, completely soft, only remnants of harder fragments may occur

*DBH* Diameter at breast height.

### Bird counts

In each of the four 30 ha areas, birds were counted using the combined cartographic method. All birds, seen and heard, were recorded excluding those flying high and not directly related to the study area^34^. Counts were performed separately in the breeding and non-breeding seasons. In the breeding season, the number of pairs was determined on the basis of the number of occupied territories identified by typical territorial and breeding behaviours of birds. During the breeding season, counts were made in the second half of May and early June 2021. Birds during the non-breeding season were counted from mid-August to the end of September 2020. Five bird counts at each site were conducted in each season. The counts were carried out in the morning hours and did not include night hours (therefore typically nocturnal birds were not included in the study). Field observations indicate that the years of the study were not mast years for beech, which is important for the numbers and species composition of the birds assemblage [e.g. ^35^].

## Data analysis

### Stand characteristics

Näslund height curves were fitted using the collected tree height data for each stand and tree species separately. The volume of living and standing entire dead trees was estimated with the use of tariff tables^36^. The volume of stumps, fallen deadwood, and snags was calculated according to Smalian’s formula. For each site, the mean values ​​of forest structural and growth indicators were calculated based on data from all 25 sample plots. However, to show variability among plot characteristics within a given stand, the results are also presented for each sample plot separately. Stand volume, density of living trees, canopy closure, sapling density, deadwood volume, and density of standing entire dead trees and snags were characterized.

The diversity of tree stand characteristics, regardless of their spatial arrangement, was assessed using the following ecological indicators:

- Shannon diversity index for assessing the species diversity of stands (Eq. 1, *p*_*i*_ – share of trees of the *i*-th species);
- Shannon diversity index for the DBH of living trees (Eq. 1) calculated on the basis of the number of trees in 9 thickness classes whose DBH ranges were sequentially: 7–16.9 cm; 17–26.9 cm; 27–36.9 cm; … 77–86.9 cm, and $\:\ge\:$ 87 cm (Eq. 1, *p*_*i*_ – share of trees in the *i*-th thickness class);
- Shannon diversity index for the DBH of standing dead trees (Eq. 1) – according to the same rules as for living trees;
- Shannon diversity index for deadwood decomposition (Eq. 1, *p*_*i*_ – share of volume of the *i*-th decay stage in total deadwood volume).
  1 $$\:H=-{\sum\:}_{i=1}^{S}\left({\text{p}}_{\text{i}}\right)\left({\text{l}\text{o}\text{g}}_{2\:}{\text{p}}_{\text{i}}\right)$$
  where: *p*_*i*_ – share of a feature in the total number or total volume, S – number of species, thickness classes, or decay stages.
- Gini index (GI) for assessing DBH diversity. GI is a measure of the concentration of a given feature and is used in ecological studies as an indicator of stand diversity (Eq. 2). Values ​​range between 0 and 1. In this case, low values of GI ​​indicate that most trees have a DBH close to the mean (small variation), while higher GI values ​​indicate greater variation.
  2 $$\:GI=\:\frac{\sum\:_{j=1}^{n}(2j-n-1){ba}_{j}}{\sum\:_{j=1}^{n}{ba}_{j}(n-1)}$$
  *j* – the rank of tree *j* in the ascending DBH series, *ba*_*j*_ – basal area of trees ranked *j* [m^2^], *n* – number of trees in the sample.

These indicators were statistically compared between individual stands. Analysis of variance and the Kruskal–Wallis test were used to determine the significance of differences. The dimensional structure of both living and dead trees as well as saplings was also evaluated. The Shapiro–Wilk test was used to assess the normality of distribution of DBH for living trees. The decay stage of the two types of deadwood and relationship with DBH was assessed. Statistical analyses were conducted using STATISTICA 13 software.

### Birds

The study analyzed the presence of birds in the breeding and non-breeding seasons separately. In the breeding season, the number of pairs was counted for each species, with population density expressed as the number of pairs per 10 ha [e.g. ^29^]. In the non-breeding season, population density was expressed as the number of individuals per 10 ha.

Similarity and diversity indices were calculated for bird communities in each of the selected stands. Actual species diversity was described using the Shannon diversity index, *H*, which takes into account the number of species and their relative abundance in the sample^37^. Species diversity measured with this index increases with the number of species as well as with greater evenness of their relative abundancies, as shown in Eq. 1, where: *p*_*i*_ is the proportion of *i*-th species in the sample and *S* is number of bird species. The Jaccard similarity index (*I*_*J*_) was computed according to the equation given below^38^ (Eq. 3):

3 $$\:Jaccard\:Index\:=\:\frac{\text{U}\text{V}}{\text{U}+\text{V}-\text{U}\text{V}}$$

where: *U* – number of species exclusively found in the first of the two compared stands, *V* – number of species exclusively found in the second of the two compared stands, *UV* – number of species found in both stands.

Additionally, to assess the type of nesting niches occupied/available, bird species in the breeding season were divided into cavity nesters, canopy nesters, and forest floor nesters (birds nesting on or near the ground) based on breeding ecology^39^, and the relative proportions of these groups in the overall bird community were determined. The species composition of bird assemblages was also illustrated using Venn diagrams—one showing the overlap among the three developmental stages of the management system, and another comparing the managed stands as a whole with the old-growth forest. Separate diagrams were provided for the breeding and non-breeding seasons.

The statistical analysis of bird assemblages used the study stands as comparison sites. Bird species were compared in each site using a statistical sign test to determine in which site a given species has higher densities. The sign test is a statistical test for consistent differences between pairs of observations. This allowed us to test whether there was a statistically significant change, due to conditions in the ecosystem, in the availability of nesting niches. These tests were performed separately for each ecological group: cavity nesters, canopy nesters, and forest floor nesters, and for the whole bird assemblage (all bird species). In addition, a statistical comparison was made between old-growth forest and the three managed stands, which were treated as part of a single management system. The mean calculated for each bird species from the three managed forest studied was used as the dependent variable. Z-statistics and p-values were provided. Statistical analyses were conducted using STATISTICA 13 software.

## Results

### Stand characteristics

**Fig. 2:**
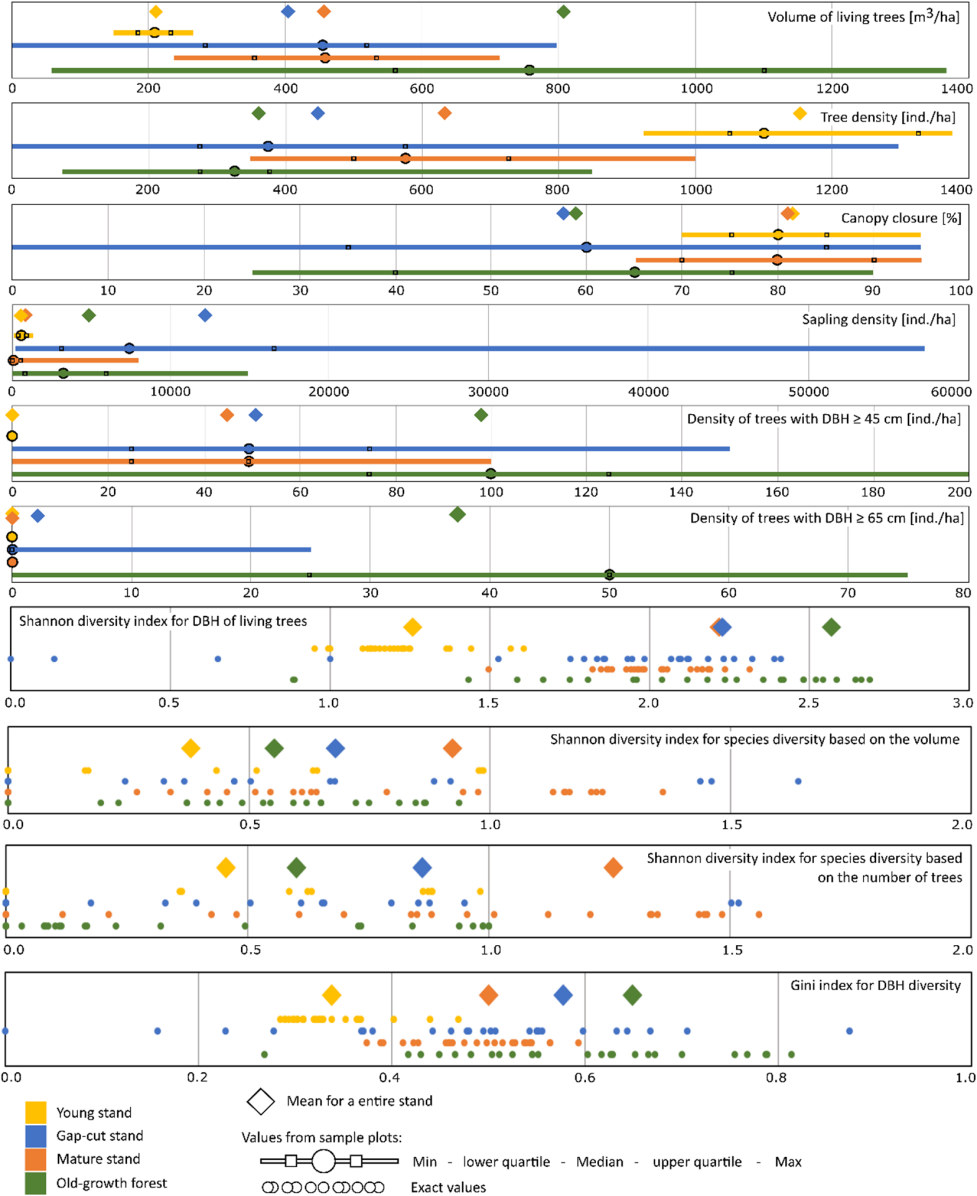
Characteristics of forest structure on individual sample plots and entire stands.

We found substantial differences in many of the measured structural characteristics both within and among the stands (Table 2). The old-growth and gap-cut stands, in particular, had strong within stand heterogeneity for all of the studied variables (Fig. 2). The volume of living trees in the old-growth stand was 807 m^3^ ha^− 1^, which was about two times higher than in the gap-cut and mature stands, and four times higher than in the young stand (Kruskal–Wallis *H* = 56.6; *p* < 0.001). The high standard errors in the Pečka Reserve and the gap-cut stand show that stand volume had high spatial variability among plots within these stands. The mean canopy cover in the Pečka Reserve and the gap-cut stand was about 60%. Both stands were similar in terms of the density of living trees and the high density of saplings (post hoc test for multiple comparisons, *p* > 0.05; Table 2). The mature and young stands, on the other hand, were fairly uniform throughout their area, with an average canopy cover about 80% and low sapling density (Fig. 2). Differences and similarities in canopy cover were confirmed by statistical tests (Kruskal–Wallis *H* = 23.5; *p* < 0.001) (Table 2).

**Table 2:** The main characteristics of the studied stands.

Variable	Pečka reserve	Mature stand	Gap-cut stand	Young stand	test	Mean
	Mean (standard error)					
Volume of living trees [m^3^ ha^−1^]	807 (70.0)a	459 (24.0)ab	403 (36.7)b	212 (6.2)c	H = 55.6 *p* < 0.001	470 (29.7)
Density of living trees [trees ha^−1^]	362 (34.2)a	630 (37.8)b	448 (58.0)ab	1155 (30.4)c	H = 25.7 *p* < 0.001	649 (37.1)
Basal area of living trees [m^2^ ha^−1^]	42 (3.3)a	37 (1.8)ab	28 (2.4)bc	26 (0.6)c	H = 65.1 *p* < 0.001	33 (1.3)
Canopy cover [%]	59 (3.9)a	81 (2.0)b	57 (5.9)a	81 (1.4)b	H = 23.5 *p* < 0.001	70 (2.2)
Sapling density [saplings ha^−1^]	4928 (845.6)a	856 (353.7)b	12,136 (3042.5)a	572 (72.5)b	H = 51.4 *p* < 0.001	4623 (912.3)
Seedlings density [individuals ha^−1^]	15,572 (2805.7)a	130,442 (18293.2)b	94,070 (17052.2)b	4513 (942.8)a	H = 60.7 *p* < 0.001	61,150 (8163.5)
Total volume of deadwood [m^3^ ha^−1^]	318 (36.9)a	20 (2.3)b	25 (3.3)b	19 (1.6)b	H = 54.1 *p* < 0.001	95 (15.8)
Volume of standing dead trees (entire and snags) [m^3^ ha^−1^]	82 (21.4)a	4 (1.3)a	1 (0.3)b	1 (0.7)ab	H = 20.0 *p* < 0.001	22 (6.3)
Density of standing dead trees (entire and snags) [trees ha^−1^]	32 (6.2)ab	44 (6.5)a	13 (4.4)b	42 (8.1)a	H = 14.5 *p* < 0.01	33 (3.4)
Volume of snags [m^3^ ha^−1^]	71 (15.8)a	1 (0.2)ab	0 (0.2)b	1 (0.6)b	H = 20.3 *p* < 0.001	18 (5.0)
Volume of stumps [m^3^ ha^−1^]	10 (2.0)a	8 (1.0)a	14 (2.8)a	9 (1.0)a	H = 2.0 *p* = 0.564	11 (0.9)
Density of stumps [stumps ha^−1^]	62 (8.9)a	231 (24.4)b	206 (24.2)b	1021 (71.4)c	H = 74.9 *p* < 0.001	380 (42.5)
Volume of downed deadwood [m^3^ ha^−1^]	226 (24.8)a	8 (1.4)b	10 (1.6)b	7 (1.0)b	H = 54.8 *p* < 0.001	62 (11.3)

H—values marked with different letters differ significantly at *p* < 0.05 as evaluated by the nonparametric Kruskal–Wallis test, corrected with a post hoc test for multiple comparisons.

While living tree density in the Pečka Reserve was low, its diameter structure was the most diverse, with large trees up to 122 cm DBH. The managed stands had higher densities with less varied diameter distributions, with trees up to 67 cm DBH in the gap-cut stand, 63 cm in the mature stand, and 42 cm in the young stand. DBH revealed a non-normal distribution (Shapiro–Wilk’s *W* = 0.988, *p* < 0.05) in all stands, characterized by a right-skewed distribution with a dominance of small trees (Appendix A, Fig. A1). In the old-growth stand there were 24 trees ha^− 1^ with a DBH above 67 cm, constituting 38% of the total volume of that stand. Differences between stands were also revealed by the Shannon and Gini diversity indices for tree size distribution. The Gini index calculated for individual sample plots shows a wide range of values, from 0 to 0.87 and from 0.27 to 0.81 for the gap-cut and the old-growth stand, respectively (Fig. 2). In the mature stand, these values ranged from 0.37 to 0.59, while in the young stand they ranged from 0.28 to 0.47. The mean values ​​calculated in total for the entire stand were also higher than in the mature and young stands. In the old-growth stand the mean Gini index was 0.65 and in the gap-cut stand it was 0.58 (Fig. 2).

**Fig. 3:**
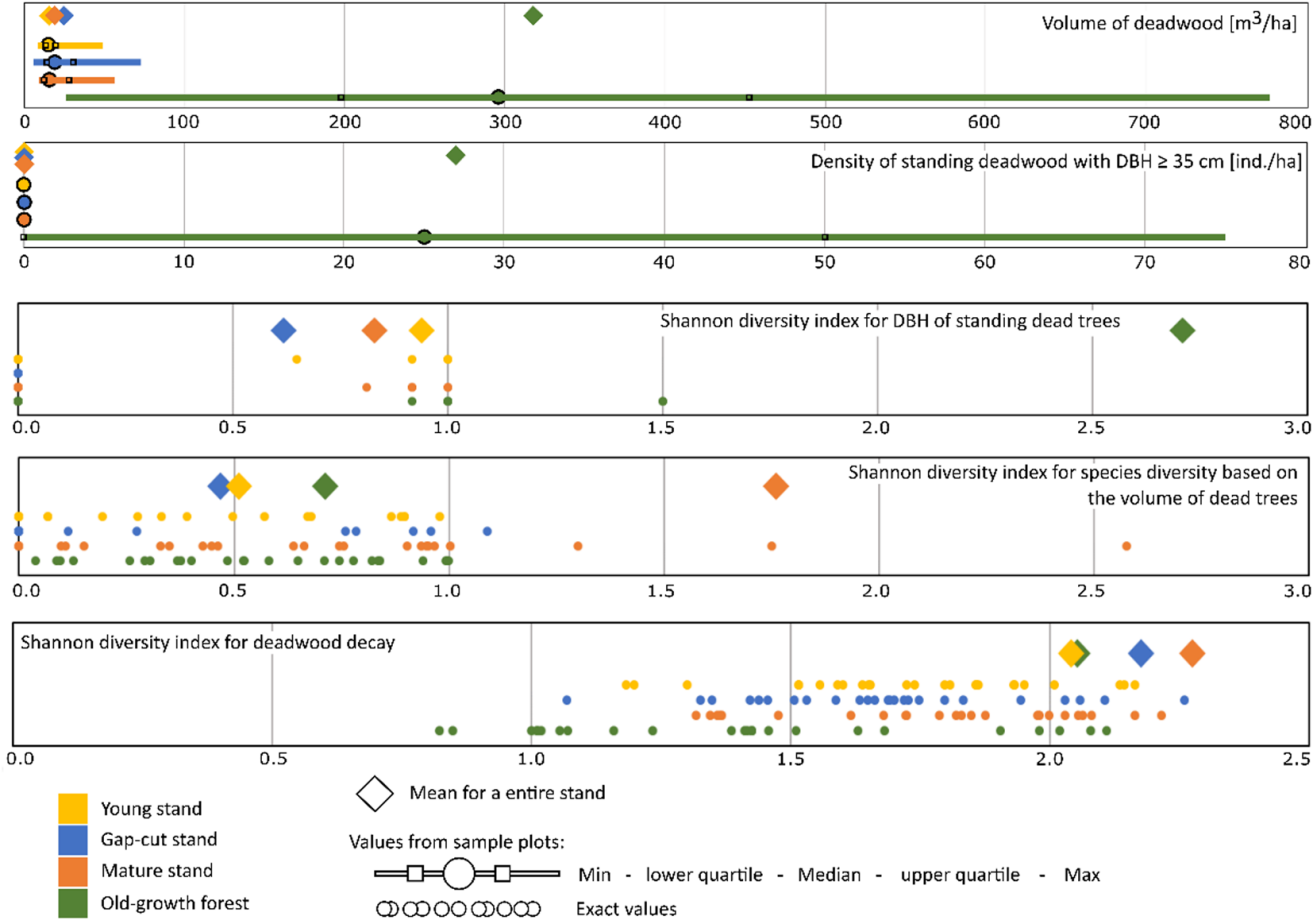
Characteristics of deadwood on individual sample plots and entire stands.

The Pečka old-growth forest differed from the other stands in that it exhibited a large amount of deadwood (Kruskal–Wallis *H* = 54.1; *p* < 0.001) at 318 m^3^ ha^− 1^ (Fig. 3). In the remaining three stands, the differences were small and not statistically significant (*p* > 0.05), with the means ranging from 19 (young stand) to 25 m^3^ ha^− 1^ (gap-cut stand). In the three managed stands, stumps accounted for the largest share of the total deadwood volume (from 41 to 58%), as compared to only 3% in the Pečka Reserve, where downed deadwood was the most abundant at 71% (226 m^3^ ha^− 1^). Even more important for birds, entire (complete) standing dead trees and snags accounted for 3% and 22% of the total deadwood volume, respectively, in the old-growth stand. The mean density of standing deadwood ranged from 13 (gap-cut stand) to 44 individuals per hectare in the mature stand. In the old-growth forests the density was 32 individuals per hectare, while in the young stand it reached 42 individuals per hectare (Table 2). Standing deadwood in the old-growth stand also exhibited a highly variable size distribution, with diameters at breast height (DBH) reaching up to 148 cm (Appendix A, Fig. A1). In the managed stands, DBH of standing deadwood did not exceed 35 cm. The Shannon index for standing deadwood DBH calculated for the entire old-growth stand was high, while in the other stands it was low. Shannon indices for deadwood decomposition were similar across the stands (Fig. 3).

### Characteristics of birds assemblages

In total, we observed 41 species during the breeding season (Pečka Reserve − 40 species, density 104.3 pairs per 10 ha; all managed forest stands combined − 33 species, mean density 56.7 pairs per 10 ha). The species that were only found in the Pečka Reserve include the Stock Dove (*Columba oenas*), White-backed Woodpecker, Middle Spotted Woodpecker (*Dendrocoptes medius*), Lesser Spotted Woodpecker (*Dryobates minor*), Red-breasted Flycatcher (*Ficedula parva*), European Crested Tit (*Lophophanes cristatus*), Willow Tit (*Poecile montanus*), and European Honey Buzzard (*Pernis apivorus*). Only one species was found only in the managed forest stands, the Tree Pipit (*Anthus trivialis*) (Appendix B, Table B1). In the non-breeding period, 27 species were recorded (Pečka Reserve – 21 species, density 45.7 ind./10 ha; managed forest stands combined – 23 species, mean density 24.3 ind./10 ha) (Appendix B, Table B2).

**Fig. 4:**
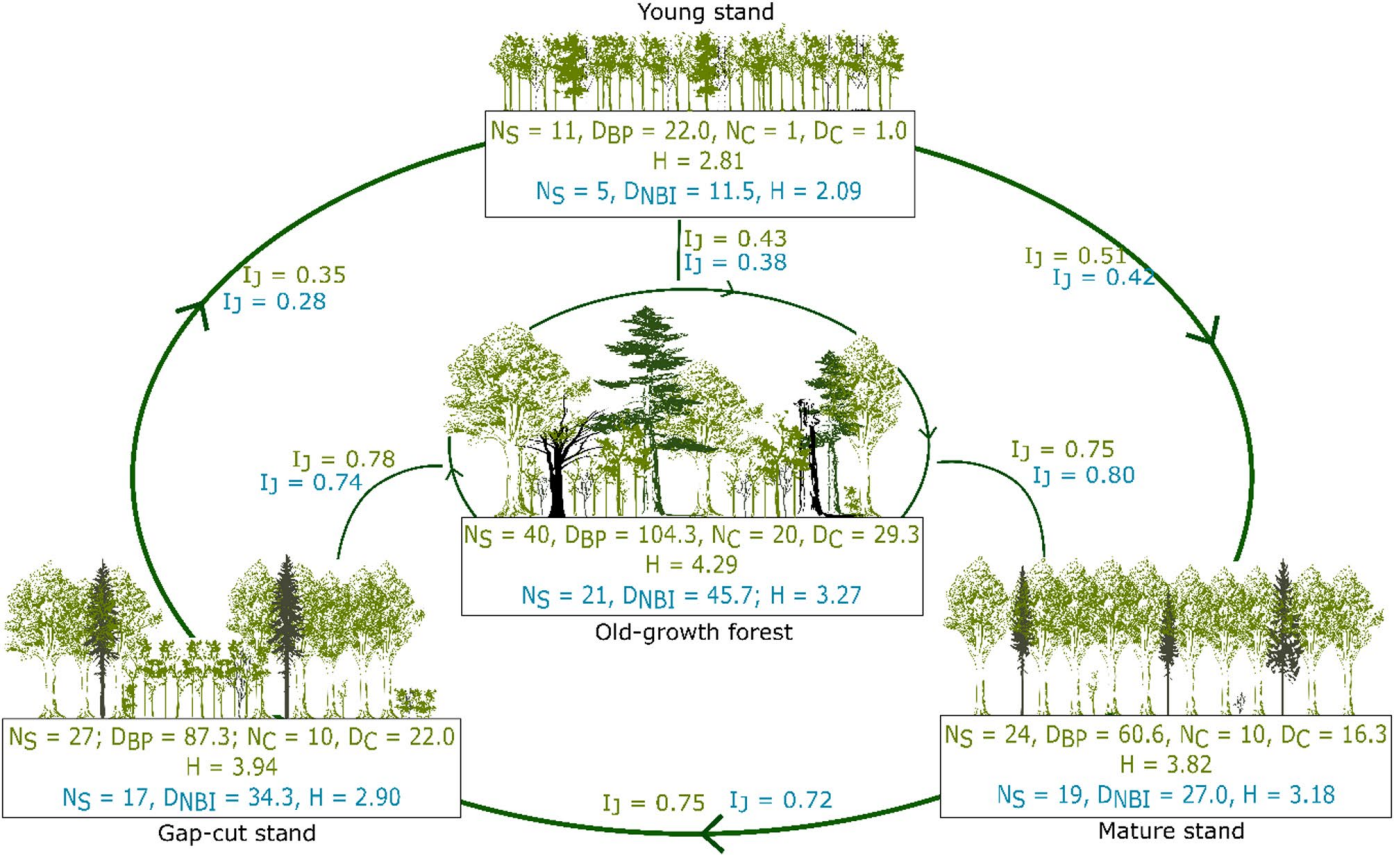
Parameters of bird assemblages in the studied stands (green color—breeding season, blue color—nonbreeding season: N_S_—number of species, D_BP_—breeding pair density, N_C_—number of cavity nesters species, D_C_—cavity nester density, H—Shannon–Wiener diversity index, D_NBI_—nonbreeding individuals density, I_J_—Jaccard Index).

**Fig. 5:**
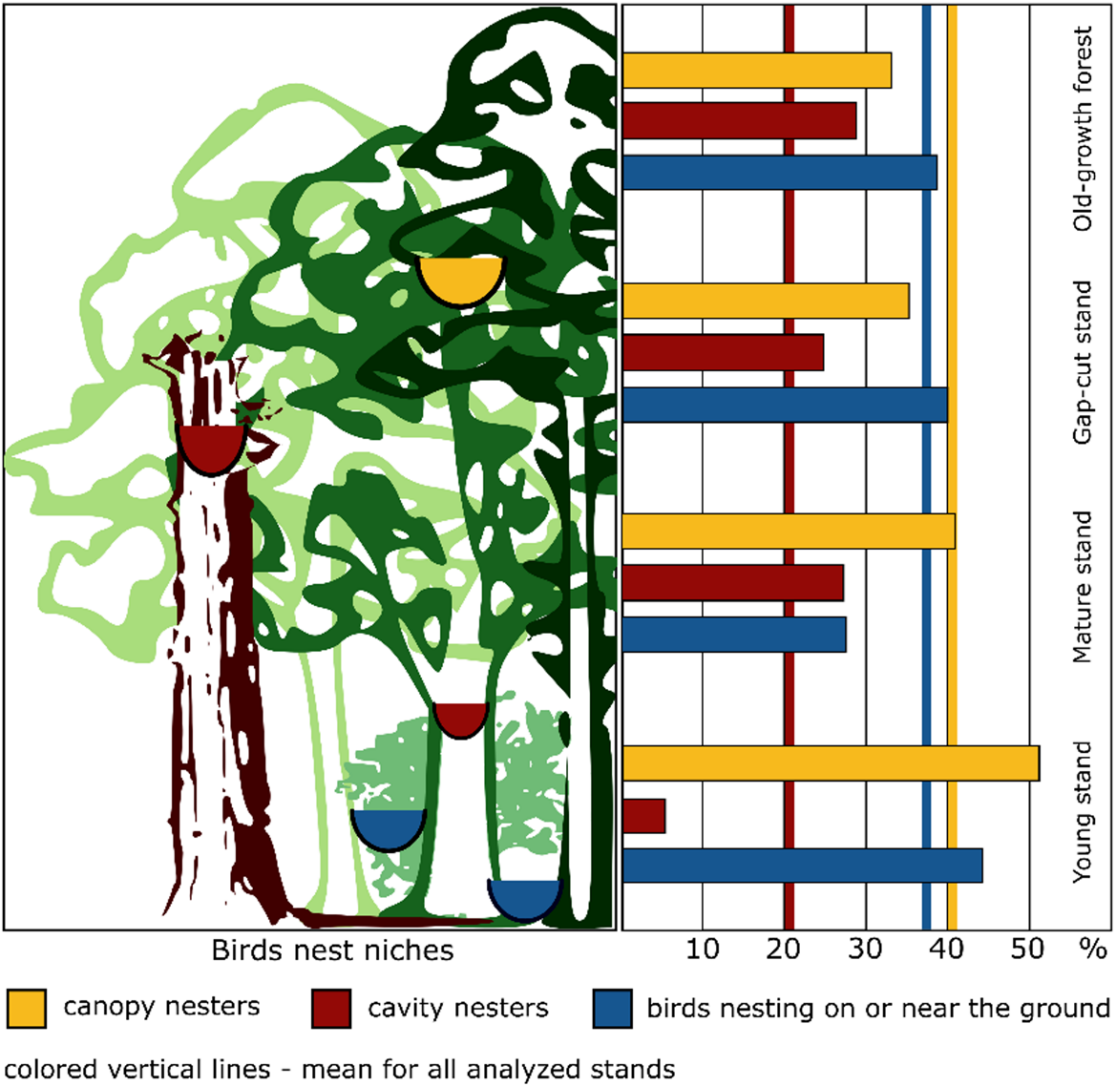
The proportion (based on densities) of birds with different nesting preferences in the four stands.

Analyzing the stands separately, in the breeding season, the Pečka Reserve had the greatest abundance of bird species (Appendix B, Table B1). Among the managed stands, the greatest number of species was found in the gap-cut stand (27 species with 87.3 pairs per 10 ha). The Shannon index was high in the old-growth stand (4.29), closely followed by the gap-cut stand (3.94). The lowest value of the index was found for the young stand (2.81). The Jaccard similarity index results were the highest between the Pečka Reserve and the gap-cut stand (0.78) and between the gap-cut stand and the mature stand (0.75). The young stand was the least similar to the other stands (Fig. 4). In terms of the share of pairs with nesting preferences, the Pečka old-growth reserve and the gap-cut stand were very similar. In both stands, a similar proportion of cavity, forest floor, and canopy nesters were observed. However, the old-growth stand had the largest share of cavity nesters (28% of breeding pairs belonging to 20 species), and the proportions between the three ecological groups were the most even. The mature and young stands were dominated by canopy nesters. Overall, cavity nesters were the least abundant. In the young stand they accounted for only 5% of the bird assemblage, represented by only one species – the Great Tit (*Parus major*) (Fig. 5). Appendix B (Figures B1 and B2) presents Venn diagrams depicting the compositional similarities and differences among the bird assemblages.

**Fig. 6:**
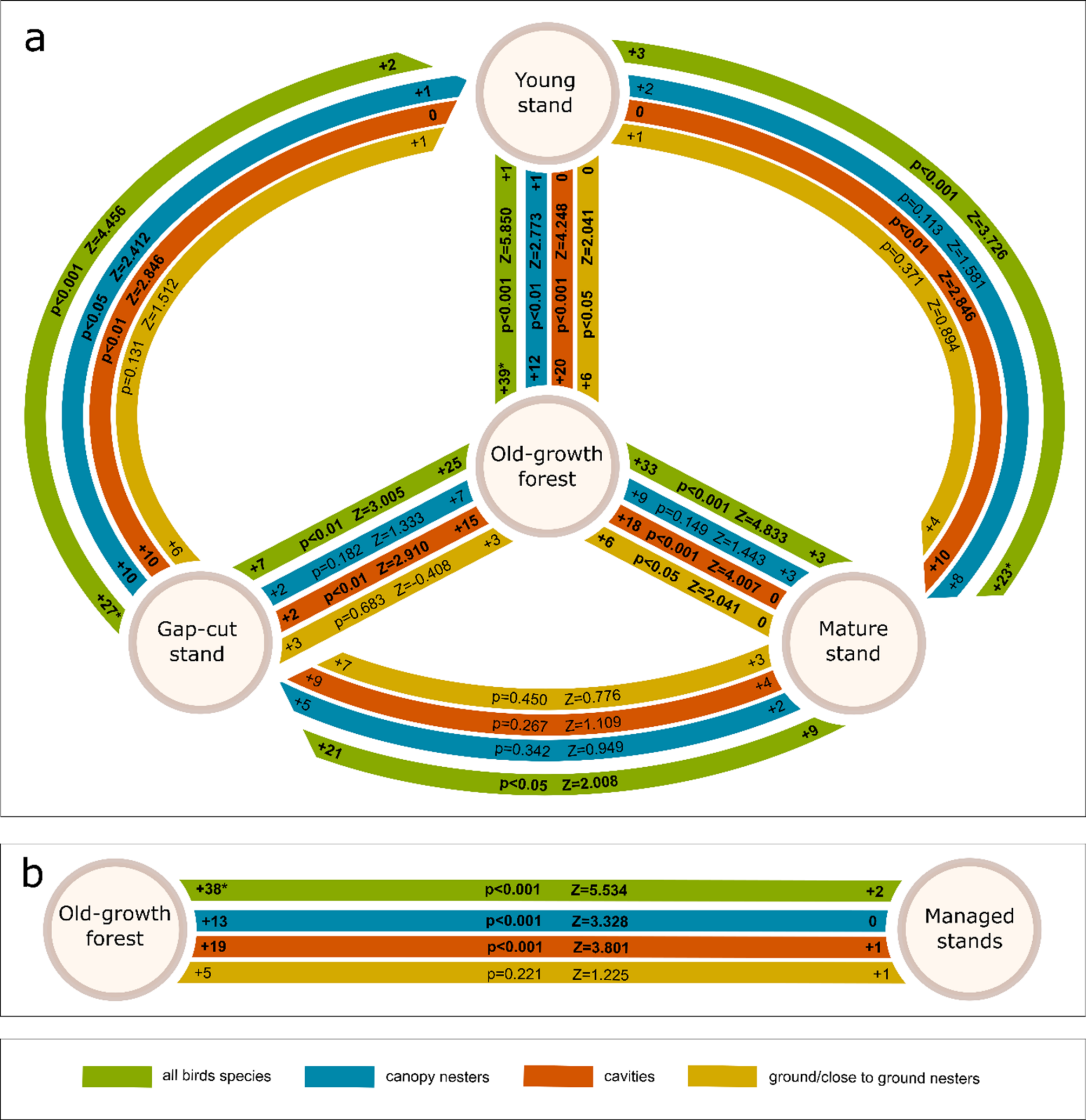
Statistical comparison (sign test) of the density of all bird species, and separately of canopy nesters, cavity nesters and birds nesting on or near the ground (**a**) between the four study stands, and (**b**) between the old-growth forest and the managed forest (in the case of the managed forest, the density of each bird species was calculated as the average of the gap-cut stand, mature stand and young stand). Numbers + 2, +5, etc. indicate how many bird species had higher densities in a given stand relative to the comparison stand. *Cuculus canorus was not assigned to any of the three groups but was included in the overall bird species density analysis. Significant differences are reported in bold letters.

Statistical tests confirmed the results described above (Fig. 6). The group with the most significant changes were the cavity nesters. When comparing the old-growth forest to other sites, the differences for this group were always statistically significant (Z-statistic value = 2.910–4.248, *p* < 0.01). Cavity nesters were statistically not different only when comparing the gap-cut stand with mature stand. For the old-growth forest, the sign test also showed differences significant between the mature stand for birds nesting on or near ground (Z = 2.041, *p* < 0.05) and in the case of the young stand for canopy nesters (Z = 2.773, *p* < 0.05). There were also statistical differences between managed stands, mainly between the young stand and the other two sites.

Comparing the old-growth forest to the mean calculated from the three managed stands, significant differences were found for canopy nesters (Z = 3.328, *p* < 0.001), cavity nesters (Z = 3.801, *p* < 0.001) and for the whole bird assemblage (Z = 5.534, *p* < 0.001). No differences occurred for birds nesting on or near ground (Fig. 6).

In the non-breeding season, the largest number of species and individuals were found in the Pečka old-growth reserve, and the lowest in the young stand (Fig. 4). Shannon diversity indices were much lower in the non-breeding season, reaching a maximum of 3.27 in the Pečka Reserve. Changes in the values of the Jaccard similarity index in relation to the breeding season were minor. Differences in species composition between the mature, gap-cut and old growth forest stands were small. Species specific to the old-growth forest were characterized by high habitat specialization, e.g. the stock dove, white-backed woodpecker, and middle spotted woodpecker.

## Discussion

The analyzed managed forests varied greatly in terms of diameter at breast height, tree volume (212–459 m^3^ ha^− 1^ Table 2), and density (448–1155 trees ha ^− 1^, Table 2), capturing different stages in the development cycle of beech forests managed with irregular shelterwood, a common close-to-nature, uneven-aged silvicultural system applied in Slovenia. The Pečka old-growth forest (stand volume 807 m^3^ ha^− 1^ and 362 trees ha ^− 1^) served as a unique reference site, distinguished by the presence of large living trees, abundant and diverse deadwood, and high structural complexity. In the studied managed forests, the maximum DBH recorded was 67 cm. In contrast, in the Pečka Reserve, the density of trees above this DBH was 24 ha^− 1^, with the total volume of these large stems amounting to 310 m^3^ ha^− 1^. Thus, a relatively small number of trees constituted 38% of the stand volume in the Pečka Reserve, which is equivalent to as much as 67% and 76% of the stand volume of the mature and gap-cut forests, respectively. The inventory of the largest primeval beech forest in Europe (Uholka-Shyrokyi Luh) showed that the maximum measured DBH was 150 cm, and there were 10 trees per hectare with a DBH of at least 80 cm^40^. These large trees represent a key difference between managed and unmanaged forests. A continuous availability of habitat trees older than 180 years and more than 20 m³ of deadwood per hectare is likely essential for maintaining high biodiversity in managed beech forests^12^; however, their presence in forest stands is strongly determined by applied silvicultural practices^41,42^. No statistically significant differences were found between the three managed forests, with the mean deadwood volume being approx. 20 m^3^ ha^− 1^, which is about 15 times less than in the Pečka old-growth forest. Importantly, in the managed forests deadwood consisted mainly of stumps and small diameter fragments. A significant reduction in deadwood amount and quality, not only at the stand but also at the landscape level, is considered to be one of the main causes of biodiversity loss in managed forest ecosystems around the world^42,43^.

In addition to the presence of large trees and deadwood, differences between the studied stands were also identified in terms of their stand-scale structure. The Pečka reserve has considerable variability in canopy structure and deadwood volume across the reserve. The mean canopy closure in the Pečka old-growth forest was 59%, but it varied considerably across individual sample plots, ranging from 25 to 90% (Fig. 2). Similarly, deadwood volume averaged 318 m³ ha⁻¹, with values ranging from 25 to 777 m³ ha⁻¹ (Fig. 3). This variability is due to a combination of individual tree mortality and windthrow events in 1983 and 2004^44^. In both storms, windthrown trees had a clumped spatial distribution, which resulted in the formation of multiple gaps of up to 1500 m^2^ in size. In addition to these events, the overall disturbance regime via gap dynamics has also contributed to the present forest mosaic. The mosaic of different size gaps throughout the stand led to a patchy, well developed layer of understory regeneration and pole sized trees^45^. Disturbance events, forming a range of different sized canopy gaps, are most likely the key processes shaping forest structure and, consequently, indirectly affecting the spatial distribution of bird assemblages^21,46–48^.

Pair densities, bird species composition and the Shannon index (ranging from 2.81 to 3.94 in managed stands and 4.29 in the old growth forest, Fig. 4) showed large differences in the richness of avifauna between the studied stands. In the Pečka Reserve, bird density was very high (more than 100 pairs per 10 ha, Fig. 4) with diversity rates comparable to other natural forests. In mountain forests, where beech is one of the main species, the Shannon index ​​ranges from 3.78 to 4.36^49–52^. Such values ​​can provide a reference for other studies on the diversity of avifauna. We suspect that the differences between the old-growth and managed forests are due to the number and type of available niches. In old-growth forests, all phases of stand development occur simultaneously over relatively small areas, from young trees recruiting in gaps to old, senescent trees undergoing death^53,54^. As a result, various ecological niches are available at all times, but in different proportions, depending on the unique history of disturbance in a given stand^55^. In managed forests, particularly in the more coarsely-scaled patches resulting from the irregular shelterwood system, some niches are less readily available and others may be missing at certain stages of forest development. Some niches develop as the stand matures, such as a higher number of tree-related microhabitats associated with larger trees^56^, or are created by overstory removal (e.g., Tree Pipit, a species associated with gaps and clearings, was found in the gap-cut forest).

In the case of mountain forests, another factor differentiating Shannon index values ​​is elevation above sea level. Lower values ​​are generally recorded at higher sites, and they tend to increase with decreasing elevation, the tree species diversity^57^. In our study, the lowest elevation was recorded at the young stand, which also supported the poorest bird assemblages, 22 pairs 10 ha^− 1^ (Fig. 4). The remaining sites were situated at similar elevations. Therefore, it can be assumed that elevation had little influence on the conclusions drawn from our research. Similar changes in diversity indices were observed in a subalpine spruce forest under strict protection, but undergoing changes as a result of tree mortality caused by insect outbreaks and windthrows^9^. In their study, following gap creation from tree mortality, and subsequent open areas with regeneration, the number of bird species, as well as the density and composition of bird assemblages changed. The Shannon index increased with the development of more diverse breeding niches^9^. Single-species beech forests are less rich in avifauna than mixed montane forests^58^. In our study, fir *(Abies alba*) or spruce (*Picea abies*) enclaves were associated with the presence of certain species, such as the Goldcrest (*Regulus regulus*), with densities ranging from 0 to 1.7 pairs per 10 ha depending on the studied stand, or the Common Firecrest (*Regulus ignicapilla*), with densities from 0 to 1.3 pairs per 10 ha (Appendix B). Consequently, the share of admixed species, mainly conifers, might have had a positive impact on index values and the number of species.

When breeding niches are relatively undifferentiated, mostly suitable only for common species which are usually associated with tree crowns, lower densities are recorded for cavity-nesters^9,59^. This was reflected in our research. The young stand offered breeding niches for 11 species (Appendix B) – mainly very common and flexible in terms of the occupied habitats, such as the European Robin (*Erithacus rubecula)*, Eurasian chaffinch (*Fringilla coelebs)*, Blackcap (*Sylvia atricapilla)* or European Blackbird (*Turdus merula*). Among them, there was one cavity nester, the Great Tit. The other two managed stands provided conditions for 10 species from the cavity nester group, in contrast to the Pečka forest with 20 species (Appendix B). The mature forest was dominated by birds nesting in crowns, while there were fewer birds nesting close to the ground, where sapling density was relatively low (Fig. 5). Conditions were apparently not improved by the small amount of deadwood, which could at least partially fill and diversify the forest floor. From a vertical perspective, it was a forest layer with few niches. In the case of the Pečka Reserve, a larger number of niches were available throughout the entire height range of the stand. Studies suggest that bird community composition and diversity increase with the complexity and variability of vertical forest structures^60–62^. Based on our research, results suggest that more complex structures are also beneficial for birds during the non-breeding period, when a greater diversity of avian communities are observed in older forests and in those offering a larger number of available niches. In temperate forests, both resident and migratory birds form mixed bird flocks. This behavior helps them avoid predation and find food [e.g., ^63^]. Nomadic flocks are constantly moving between stands, and it is obvious that they will be found more often in forests that are richer in food sources. In the Pečka Reserve, 21 species were recorded with a mean density of over 45 individuals per 10 ha, but it is possible that in beech mast years the number and diversity of birds could be higher^35^, unless pressure from predators also intensifies during mast years^64^. In the young stand, only 5 species were identified with a mean density of 11 individuals per 10 ha. The trees in that stand were still too young to produce seeds, and so the number of non-breeding birds would not increase even in years of abundant beech mast production.

One of the key findings of our study was that, in addition to supporting a high number of birds, the Pečka Reserve provides breeding sites for rare species that are less flexible in terms of habitat selection and have narrower ecological niches (e.g., Stock Dove, White-backed Woodpecker, Middle Spotted Woodpecker, and some owl species such as the Ural Owl (*Strix uralensis*); Appendix B). The presence of old trees and standing deadwood significantly enhances their nesting opportunities and may play a crucial role in preserving populations of forest specialist birds. Various studies have indicated a relationship between stand age and bird diversity. Based on data for breeding birds, mollusks and lichens, Moning and Müller^65^ identified significant forest age threshold ranges for the occurrence of these old-growth sensitive taxa. In all three taxonomic groups the number of species per plot significantly increases with forest age. In mixed montane forests threshold values range from 160 to 220 years. These values are ​​above the rotation ages used in managed forests. This is why old-growth forests or large old trees retained in managed forests are so important for bird diversity. They create ecological niches that do not exist at all or occur rarely in younger forests, such as abundant cavities (including large cavities), dead parts of standing trees, reservoirs with rainwater in the cavities of trunks and branches, rough bark, and many other unique microhabitats [e.g., ^18,56,66–68^]. Downed deadwood volume in Pečka was 226 m³ ha⁻¹, while in the managed stands it ranged from 7 to 10 m³ ha⁻¹. In Pečka, it accounted for over 70% of the total deadwood volume, compared to approximately 40% in the managed stands (Table 2). Although downed deadwood is a less important structural component for nesting, it is a highly important feeding habitat for some species, such as the White-backed Woodpecker, which feeds on the larvae of saproxylic beetles^69,70^. Similar conclusions follow from other comparative works on old-growth and managed forests in the Dinaric region of Slovenia. A similar number of species and overall bird density, more cavity-nesting birds, more species feeding on and under bark, more woodpigeons, and many more rare and endangered species have been found in old-growth forest remnants^21,22,71^.

In light of the presented differences, a fundamental question is to what extent managed forests can provide the conditions for sustaining viable and stable populations of species associated with forest ecosystems^72^. Is the use of close-to-nature silviculture sufficient to preserve the most sensitive species? This method involves the use of native species, natural regeneration, and relatively fine-grained forest management systems, while protecting soils that are sensitive to erosion. Tree harvesting may partially imitate natural processes, ensuring the continuity of forest canopy cover^24–26^. In the present study, cuts initiating regeneration led to differences in forest structure and therefore the gap-cut stand was the most similar to the old-growth forest. The remaining stands (mature and young) were much less diverse and more spatially uniform, which resulted in a significant reduction in the range of available ecological niches. However, all three of the managed stands should be treated as part of a single management system, acting as a shifting mosaic on the landscape^3,73^. Therefore, we should examine the managed forest in a landscape scale rather than a stand scale. It is therefore especially important to identify the elements that are missing at the landscape scale, the absence of which may have an adverse effect on the presence of certain bird species.

## Conclusions

Improving habitat conditions for birds can be achieved by retaining large living trees above certain DBH thresholds^74,75^. This can be gradually achieved at the landscape scale, starting with stands currently undergoing final harvesting operations. By retaining individual trees or small patches of stands, comprising a small proportion of the harvested area, it is possible to facilitate this process over longer time scales. Such trees would reach their maximum biological lifespan and subsequently provide microhabitats associated with habitat trees and ultimately deadwood. In some cases, active creation of deadwood and snags can also be encouraged to more rapidly reach desired targets. It should be also noted that rotation age is one of the simplest but ecologically salient variables ​​that may be controlled by forest management. When shelterwood systems with a long regeneration period are used, mountain forests are often of different ages over large scales. A greater abundance of bird species seems to be associated with stand features that are typical of old-growth, such as rough bark, large amounts of deadwood, nesting cavities, and large trees^76,77^. In conclusion, our research indicates that commercial forests as a whole provide many niches for nesting birds. However, compared to the old growth forest, the average densities of most bird species are lower. Therefore, identifying important micro-habitats for birds and making adjustments to forest management may, for many species, raise their average densities.

## Supplementary Information

Below is the link to the electronic supplementary material.

Supplementary Material 1

Supplementary Material 2

## Data Availability

Some of the data are included in the appendices to this paper. However, the data on stands are deposited and can be viewed on servers at the University of Agriculture in Cracow, Faculty of Forestry, Department of Forest Biodiversity. Contact point: Małgorzata Bujoczek; malgorzata.bujoczek@urk.edu.pl.
