# Supplementary material for: Effects of continuous cover management on bird communities in a beech dominated forest region of Slovenia
Source: Sci Rep. 2025 Sep 29;15:33614. doi: 10.1038/s41598-025-19071-x (PMC12479758; doi:10.1038/s41598-025-19071-x)
Supplement: Supplementary file 1 — Supplementary Material 1 [file 41598_2025_19071_MOESM1_ESM.docx]

Appendix A


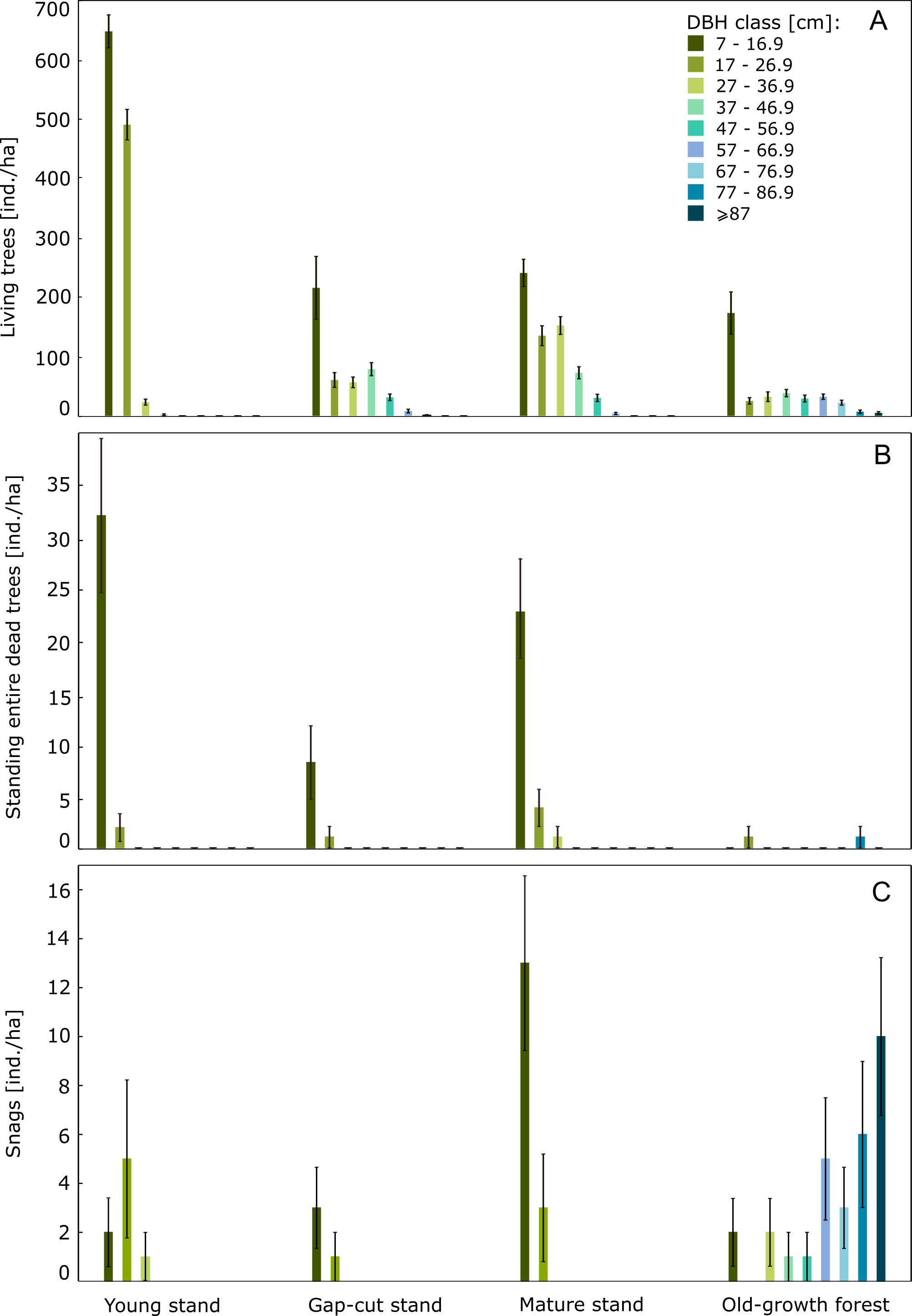


Fig. A1. DBH distribution of living trees (A), standing entire (complete) dead trees (B), and snags (C). Box – mean value, whiskers – standard error

Living trees:

Young stand – Fagus sylvatica (93,2% share in density, 92,0% in volume), Abies alba (0,0%, 0.0%), Picea abies (6.4%, 7.0%), others (total 0.3%, 1.0%);

Mature stand – Fagus sylvatica (82.9% share in density, 71.3% in volume), Abies alba (0.0%, 0.0%), Picea abies (6.7%, 14.3%), others (total 10.4%, 14.4%);

Gap-cut stand – Fagus sylvatica (89.6% share in density, 84.8% in volume), Abies alba (0.4%, 0.1%), Picea abies (4.2%, 4.3%), others (total 5.8%, 10.8%);

Old-growth forest reserve Pečka – Fagus sylvatica (87.8% share in density, 85.3% in volume), Abies alba (11.9%, 14.6%), Picea abies (0.3%, 0.1%), others (total 0.0%, 0.0%);

Standing entire dead trees:

Young stand – Fagus sylvatica (64.7% share in density, 83.5% in volume), Abies alba (0.0%, 0.0%), Picea abies (35.3%, 16.5%), others (total 0.0%, 0.0%);

Mature stand – Fagus sylvatica (53.6% share in density, 16.1% in volume), Abies alba (0.0%, 0.0%), Picea abies (28.6%, 19.7%), others (total 17.8%, 64.2%);

Gap-cut stand – Fagus sylvatica (77.8% share in density, 60.4% in volume), Abies alba (0.0%, 0.0%), Picea abies (11.1%, 3.8%), others (total 11.1%, 35.8%);

Old-growth forest reserve Pečka – Fagus sylvatica (0.0% share in density, 0.0% in volume), Abies alba (100.0%, 100.0%), Picea abies (0.0%, 0.0%), others (total 0.0%, 0.0%);

Snags:

Young stand – Fagus sylvatica (100.0% share in density, 100.0% in volume), Abies alba (0.0%, 0.0%), Picea abies (0.0%, 0.0%), others (total 0.0%, 0.0%);

Mature stand – Fagus sylvatica (87.5% share in density, 75.6% in volume), Abies alba (0.0%, 0.0%), Picea abies (0.0%, 0.0%), others (total 12.5%, 24.4%);

Gap-cut stand – Fagus sylvatica (100.0% share in density, 100.0% in volume), Abies alba (0.0%, 0.0%), Picea abies (0.0%, 0.0%), others (total 0.0%, 0.0%);

Old-growth forest reserve Pečka – Fagus sylvatica (30.0% share in density, 14.2% in volume), Abies alba (70.0%, 85.8%), Picea abies (0.0%, 0.0%), others (total 0.0%, 0.0%);
