# Supplementary material for: Effects of continuous cover management on bird communities in a beech dominated forest region of Slovenia
Source: Sci Rep. 2025 Sep 29;15:33614. doi: 10.1038/s41598-025-19071-x (PMC12479758; doi:10.1038/s41598-025-19071-x)
Supplement: Supplementary file 2 — Supplementary Material 2 [file 41598_2025_19071_MOESM2_ESM.docx]

Appendix B

Table B1. Breeding birds communities in different stands

| Species | Old-growth forest reserve Pečka | Gap-cut stand | Mature stand | Young stand |
| --- | --- | --- | --- | --- |
|  | pairs/10 ha | | | |
| *Aegithalos caudatus* | 0,7 | 0,3 | 0,0 | 0,0 |
| *Anthus trivialis* | 0,0 | 0,3 | 0,0 | 0,0 |
| *Buteo buteo* | 0,3 | 0,3 | 0,3 | 0,0 |
| *Certhia familiaris* | 3,0 | 2,0 | 2,3 | 0,0 |
| *Coccothraustes coccothraustes* | 0,3 | 0,0 | 0,0 | 0,5 |
| *Columba oenas* | 2,0 | 0,0 | 0,0 | 0,0 |
| *Columba palumbus* | 1,3 | 1,0 | 0,3 | 0,0 |
| *Cuculus canorus* | 0,7 | 0,7 | 0,7 | 0,5 |
| *Cyanistes caeruleus* | 1,0 | 1,0 | 0,7 | 0,0 |
| *Dendrocopos leucotos* | 0,3 | 0,0 | 0,0 | 0,0 |
| *Dendrocopos major* | 1,3 | 1,0 | 0,7 | 0,0 |
| *Dendrocoptes* *medius* | 0,3 | 0,0 | 0,0 | 0,0 |
| *Dryobates minor* | 0,3 | 0,0 | 0,0 | 0,0 |
| *Dryocopus martius* | 0,7 | 0,3 | 0,0 | 0,0 |
| *Erithacus rubecula* | 12,3 | 9,0 | 4,7 | 7,0 |
| *Ficedula albicollis* | 1,0 | 0,0 | 0,3 | 0,0 |
| *Ficedula parva* | 0,3 | 0,0 | 0,0 | 0,0 |
| *Fringila coelebs* | 11,7 | 9,7 | 12,3 | 5,0 |
| *Garrulus glandarius* | 1,3 | 1,3 | 1,0 | 0,0 |
| *Lophophanes cristatus* | 0,3 | 0,0 | 0,0 | 0,0 |
| *Muscicapa striata* | 0,7 | 0,0 | 0,3 | 0,0 |
| *Oriolus oriolus* | 0,3 | 0,3 | 0,0 | 0,0 |
| *Parus major* | 7,0 | 6,7 | 5,3 | 1,0 |
| *Periparus ater* | 4,7 | 6,0 | 4,0 | 0,0 |
| *Pernis apivorus* | 0,3 | 0,0 | 0,0 | 0,0 |
| *Phyloscopus colybita* | 3,3 | 9,0 | 1,0 | 0,0 |
| *Phyloscopus sibilatrix* | 2,7 | 0,0 | 0,7 | 0,5 |
| *Picus canus* | 0,3 | 0,3 | 0,0 | 0,0 |
| *Poecile montanus* | 0,7 | 0,0 | 0,0 | 0,0 |
| *Poecile palustris* | 0,3 | 1,3 | 0,3 | 0,0 |
| *Prunella modularis* | 0,3 | 0,3 | 0,0 | 0,0 |
| *Regulus ignicapilla* | 0,7 | 0,0 | 1,3 | 0,0 |
| *Regulus regulus* | 1,3 | 1,7 | 0,0 | 0,5 |
| *Sitta europaea* | 4,3 | 3,0 | 2,0 | 0,0 |
| *Strix aluco* | 0,3 | 0,3 | 0,0 | 0,0 |
| *Strix uralensis* | 0,3 | 0,0 | 0,3 | 0,0 |
| *Sylvia atricapilla* | 9,0 | 11,7 | 3,0 | 2,0 |
| *Troglotydes troglotydes* | 12,0 | 4,3 | 6,7 | 0,0 |
| *Turdus merula* | 10,7 | 9,0 | 6,7 | 3,0 |
| *Turdus philomelos* | 4,7 | 5,3 | 4,3 | 1,5 |
| *Turdus viscivorus* | 1,0 | 1,0 | 1,3 | 0,5 |

Table B2. Non-breeding birds communities in different stands

| Species | Old-growth forest reserve Pečka | Gap-cut stand | Mature stand | Young stand |
| --- | --- | --- | --- | --- |
|  | ind./10 ha | | | |
| *Certhia familiaris* | 1,7 | 0,5 | 0,7 | 0,0 |
| *Corvus corax* | 0,3 | 0,0 | 0,3 | 0,0 |
| *Columba oenas* | 0,5 | 0,0 | 0,0 | 0,0 |
| *Cyanistes caeruleus* | 1,3 | 2,3 | 1,5 | 2,0 |
| *Dendrocopos leucotos* | 0,5 | 0,0 | 0,0 | 0,0 |
| *Dendrocopos major* | 0,3 | 0,3 | 0,3 | 0,0 |
| *Dendrocoptes* *medius* | 0,3 | 0,0 | 0,0 | 0,0 |
| *Erithacus rubecula* | 2,7 | 2,7 | 0,2 | 0,0 |
| *Fringila coelebs* | 1,5 | 0,2 | 0,3 | 0,0 |
| *Garrulus glandarius* | 1,0 | 0,0 | 0,3 | 0,0 |
| *Lophophanes cristatus* | 0,0 | 0,0 | 0,3 | 0,0 |
| *Loxia curvirostra* | 0,0 | 0,0 | 0,3 | 0,0 |
| *Parus major* | 13,0 | 15,2 | 9,0 | 5,0 |
| *Periparus ater* | 4,2 | 0,5 | 3,0 | 0,0 |
| *Phyloscopus colybita* | 0,2 | 3,2 | 0,0 | 0,0 |
| *Phyloscopus sibilatrix* | 0,3 | 0,0 | 1,2 | 0,0 |
| *Picus canus* | 0,3 | 0,0 | 0,0 | 0,0 |
| *Poecile montanus* | 0,3 | 0,2 | 0,2 | 0,0 |
| *Poecile palustris* | 0,0 | 0,0 | 0,2 | 0,0 |
| *Prunella modularis* | 0,0 | 0,2 | 0,0 | 0,0 |
| *Regulus regulus* | 9,7 | 2,7 | 3,8 | 2,0 |
| *Sitta europaea* | 4,8 | 2,3 | 3,3 | 1,0 |
| *Sylvia atricapilla* | 0,0 | 0,3 | 0,0 | 0,0 |
| *Troglotydes troglotydes* | 2,0 | 0,3 | 0,2 | 0,0 |
| *Turdus merula* | 0,5 | 1,3 | 1,3 | 1,5 |
| *Turdus philomelos* | 0,0 | 1,7 | 0,0 | 0,0 |
| *Turdus viscivorus* | 0,2 | 0,5 | 0,5 | 0,0 |


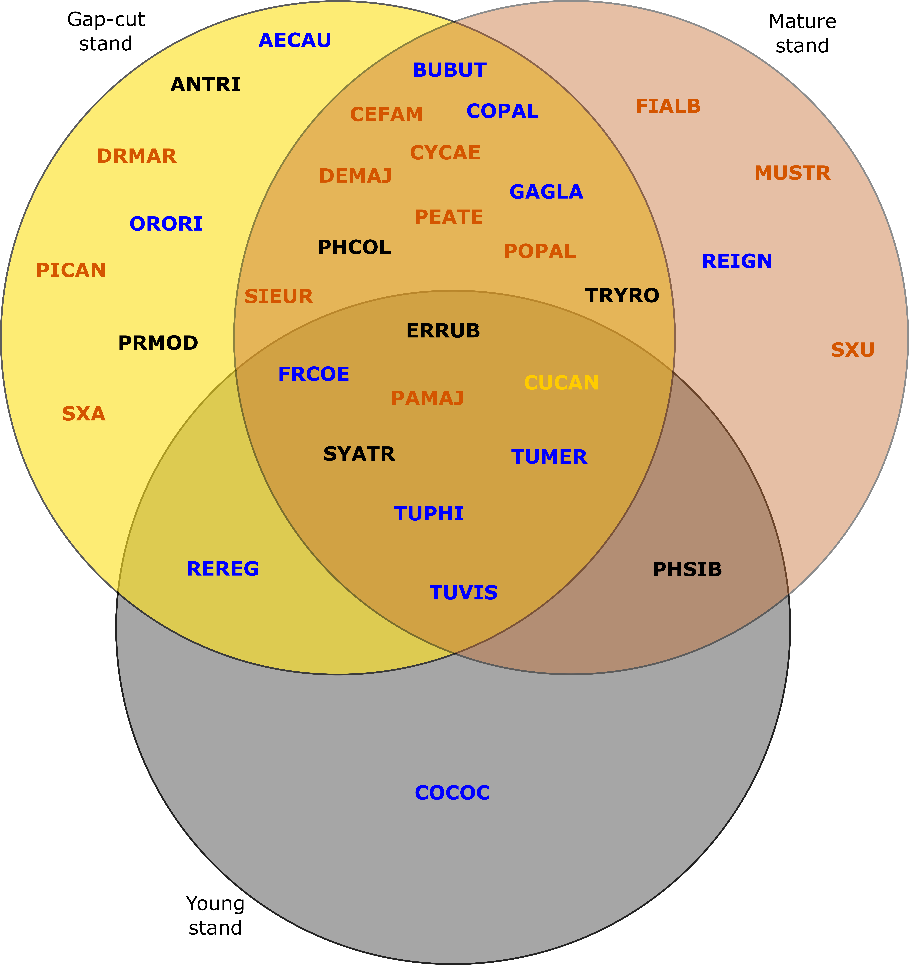


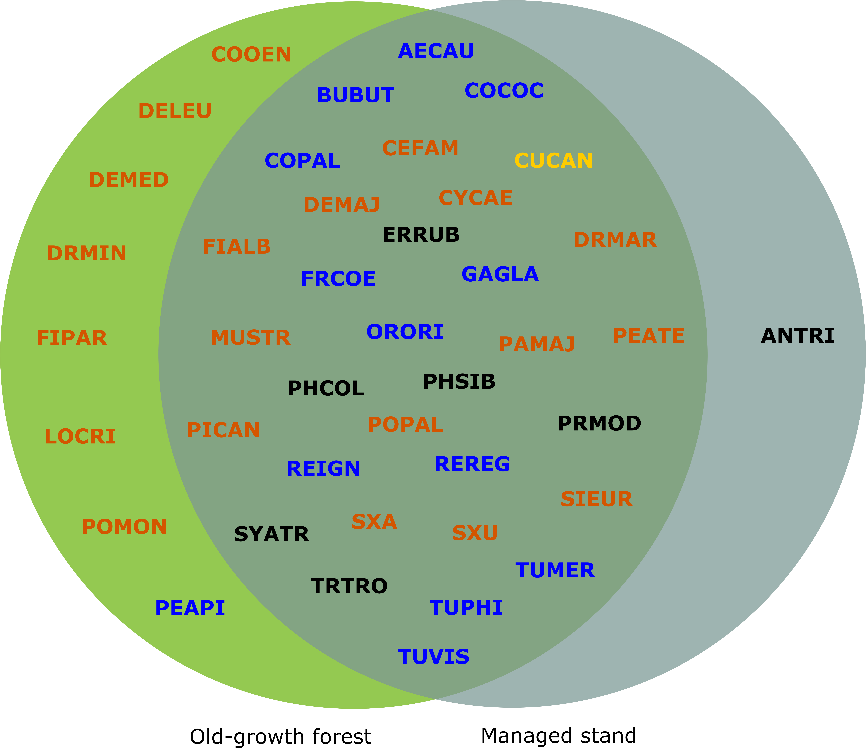


Fig. B1. Breeding birds species found in different types of stands and common to them

AECAU *Aegithalos caudatus*, ANTRI *Anthus trivialis*, BUBUT *Buteo buteo*, CEFAM *Certhia familiaris*, COCOC *Coccothraustes coccothraustes*, COOEN *Columba oenas*, COPAL *Columba palumbus*, CO COR *Corvus corax* CUCAN Cuculus canorus, CYCAE Cyanistes caeruleus, DELEU *Dendrocopos leucotos,* DEMAJ *Dendrocopos major,* DEMED *Dendrocoptes* *medius,* DRMIN *Dryobates minor,* DRMAR *Dryocopus martius,* ERRUB *Erithacus rubecula,* FIALB *Ficedula albicollis,* FIPAR *Ficedula parva,* FRCOE *Fringila coelebs,* GAGLA *Garrulus glandarius,* LOCRI *Lophophanes cristatus,* LOCUR *Loxia curvirostra,* MUSTR *Muscicapa striata,* ORORI *Oriolus oriolus,* PAMAJ *Parus major,* PEPAR *Periparus ater,* PEAPI *Pernis apivorus,* PHCOL *Phyloscopus colybita,* PHSIB *Phyloscopus sibilatrix,* PICAN *Picus canus,* POMON *Poecile montanus,* POPAL *Poecile palustris,* PRMOD *Prunella modularis,* REIGN *Regulus ignicapilla,* REREG *Regulus regulus,* SIEUR *Sitta europaea,* SXA *Strix aluco,* SXU *Strix uralensis,* SYATR *Sylvia atricapilla,* TRTRO *Troglotydes troglotydes,* TUMER *Turdus merula,* TUPHI *Turdus philomelos,* TUVIS *Turdus viscivorus*


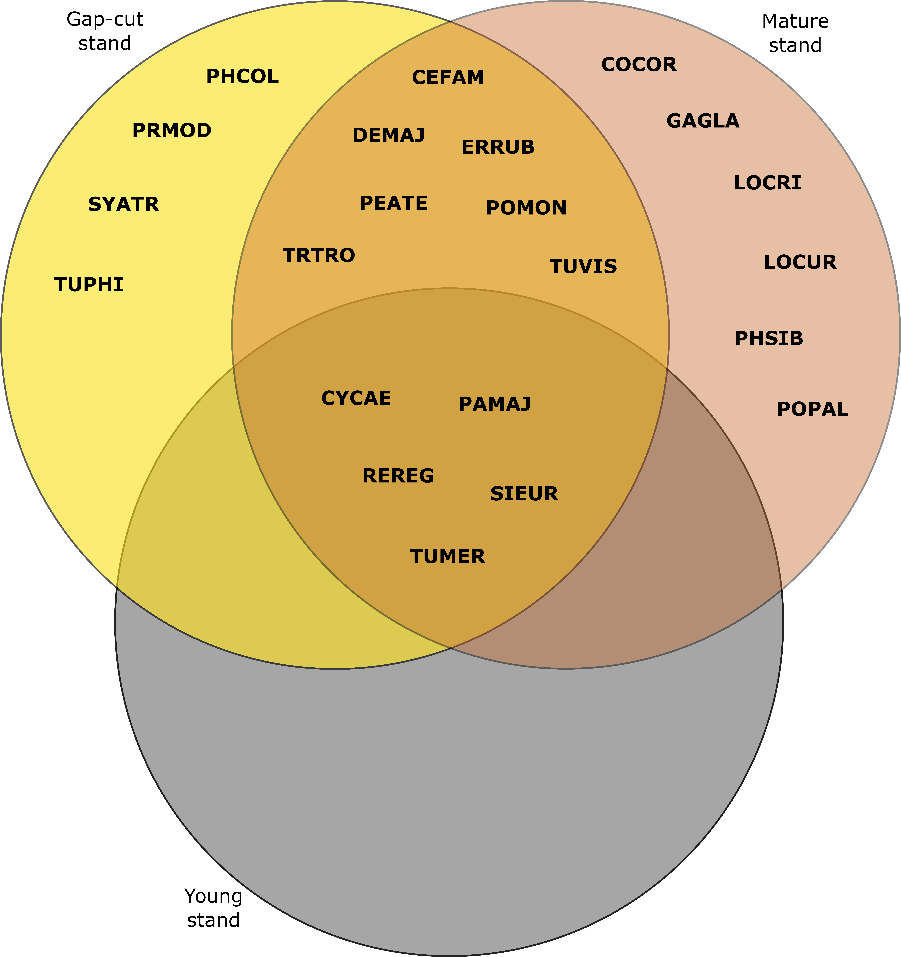


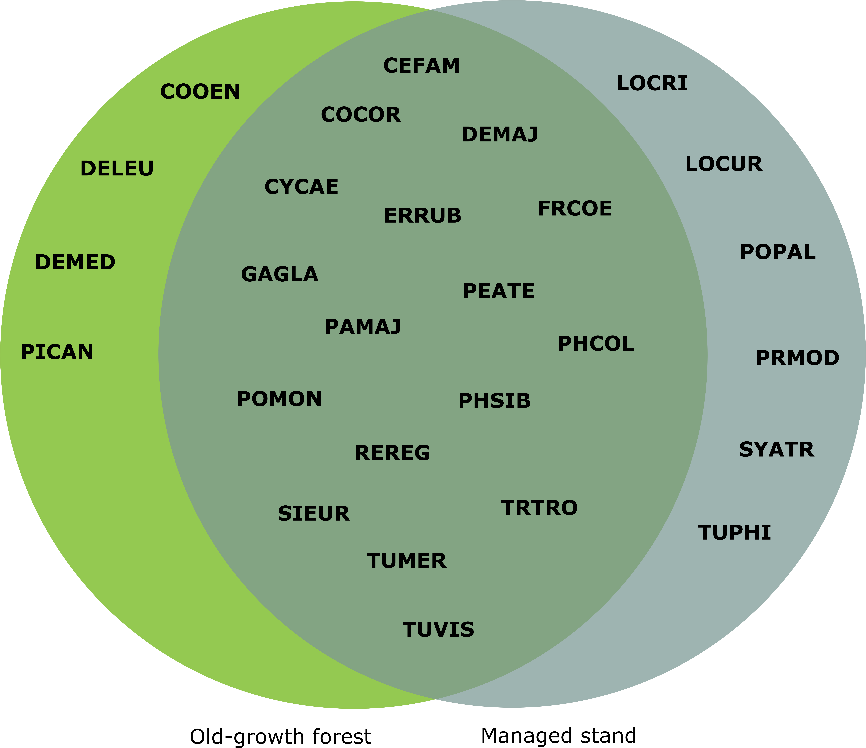


Fig. B2. Nonbreeding birds species found in different types of stands and common to them (explanations as in the figure B1)
